# Epidemiology of *Staphylococcus pseudintermedius* in cats in Poland

**DOI:** 10.1038/s41598-021-97976-z

**Published:** 2021-09-23

**Authors:** K. Bierowiec, M. Miszczak, A. Korzeniowska-Kowal, A. Wzorek, D. Płókarz, A. Gamian

**Affiliations:** 1 Division of Infectious Diseases and Veterinary Administration, Department of Epizootiology and Clinic of Birds and Exotic Animals, Faculty of Veterinary Medicine, Wroclaw University of Environmental and Life Sciences, 50-366 Wroclaw, Poland; 2 Department of Immunology of Infectious Diseases, Hirszfeld Institute of Immunology and Experimental Therapy, Weigla 12, 53-114 Wroclaw, Poland

**Keywords:** Antimicrobials, Bacteriology, Infectious-disease diagnostics

## Abstract

*Staphylococcus pseudintermedius* is a well-known coagulase-positive staphylococcus that is mainly associated with the asymptomatic colonization of the skin of pets and mucous membranes. Little is still known about the occurrence of *S. pseudintermedius* in cats. The current study aimed to characterize the isolates of *S. pseudintermedius* from sick and healthy cats. This was achieved by examining their antibiotic resistance properties, biofilm formation, and genotype differences. Six hundred and seventy-six cats were swabbed (595 healthy and 81 sick cats). Thirty-five distinct *S. pseudintermedius* isolates from 27 cats were isolated. The prevalence of *S. pseudintermedius* in healthy and sick cats was 2.49% and 7.61%, respectively. In comparison, MRSP (methicillin-resistant *Staphylococcus pseudintermedius*) prevalence was 0.12% and 2.98%, respectively. Cats were more frequently colonized with *S. pseudintermedius* when kept with dogs, regardless of their health condition, with this result being statistically significant. Multidrug resistance was detected in 50%, and 38.46% of *S. pseudintermedius* isolates from healthy and sick cats, respectively. In contrast, genetic multidrug resistance was detected in 59% and 46.15% cases, respectively. Seven from eight isolated MRSPs were multidrug-resistant. Multi-locus sequence typing (MLST) assigned isolates to 19 types, of which 16 types submitted for the first time to the PubMLST database. The most frequently detected STs (sequence types) were 551 and 71. ST71 and ST551 were mainly isolated from cats with clinical signs of infection. All were MRSPs, regardless of cats’ health. These isolates were characterized with the most frequent antibiotic resistance at the phenotypic and genotypic level.

## Introduction

The skin and mucous membranes of mammals are sources of opportunistic pathogens; however, the role of those microbiomes in health and disease remains poorly understood. Assessment of microbiota in felines is particularly limited. Consequently, there is a paucity of knowledge on how certain bacterial species have adapted to feline hosts. Some reports showed that *Staphylococcus pseudintermedius (S. pseudintermedius)* is less adapted to adhering to feline corneocytes compared to canine corneocytes in vitro^1^. This phenomenon might explain the much lower prevalence rate of *S. pseudintermedius* colonization in cats compared to dogs (0–36.84% versus 24.4–92%, respectively)^2–6^. *S. pseudintermedius* is the most frequent cause of pyoderma in dogs^7,8^. It is also often isolated from veterinary patients with infected wounds, surgical sites, external ear canal, respiratory, or urogenital tracts^9–12^. *S. pseudintermedius* is also isolated from cats with respiratory tract infections, conjunctivitis, urogenital infections, dermatitis, otitis, and wounds. These pathological changes are mostly caused by methicillin-resistant *S. pseudintermedius* (MRSP)^13–15^. Factors that potentially increase the risk of *S. pseudintermedius* infection have been described for dogs and cats. *S. pseudintermedius* infection has been related to animals with previous hospitalization or frequent visits to veterinary settings, along with the administration of glucocorticosteroids and antibacterial chemotherapeutics^12,14^.

Previously^15^, we described a statistically significant connection between the more frequent presence of *S. pseudintermedius* and the state of health of investigated cats. As an extension of that study, here, we explore the colonization characteristics of *S. pseudintermedius* in cats. We hypothesized the following: (1) it might form part of the natural microbiota in cats; (2) it might arise through pathological changes in cats; or (3) its presence in healthy cats is connected to close contact with dogs or other favorable factors, particularly as it is the most frequently isolated coagulase-positive *Staphylococcus* (CPS) in dogs. To explore these possibilities, we characterized *S. pseudintermedius* isolates collected from both healthy and sick cats. We then statistically analyzed risk factors potentially associated with the colonization of *S. pseudintermedius* in both groups of cats.

## Materials and methods

### Study population and sampling procedures

Samples were collected between 2013 and 2019 from patients at veterinary clinics in the Wrocław City area, Poland. Swabs were collected from healthy cats and other animals with suspicion of bacterial infection of the upper respiratory tract, skin/wounds, or conjunctiva. Swabs were collected from 595 healthy and 81 sick cats. Most cats were housekeeping pets, but some samples were collected from free-living cats sampled during a trap, neuter, and release (TNR) program for the humane control of the feral cat population. Four swabs were collected from each animal; specifically, the conjunctival sac, nares, skin of the groin, and anus. An additional swab was also collected from changed skin or wounds of cats in the sick group. During the collection of swabs, cat owners were asked to complete a questionnaire on the health of their cat, along with potential risk factors connected with staphylococci. This questionnaire included questions about animal features, such as age, sex, breed, and medical history. Household factors included medical occupation or previous hospitalization of household members, other animals kept in the same household and their medical history.

The research project was submitted to the Local Ethics Committee for Animal Experiments in Wrocław, Hirszfeld Institute of Immunology and Experimental Therapy, Polish Academy of Sciences. Due to noninvasive sampling, the Ethics Committee qualified the study as research, thereby exempting it from any further approval from the Ethics Committee. All methods described were approved by Wroclaw University of Environmental and Life Sciences and were performed in compliance with the relevant guidelines and regulations for good laboratory practice. Each cat owner submitted informed consent to take part in this study and filled out the proper documentation.

### Species identification

Collected swabs were placed in 2 ml of liquid brain–heart infusion broth (BHI) (Oxoid, Basingstoke, United Kingdom), and were incubated at 37 °C for 24 h. The material was then subcultured onto solid media, and incubated for another 24 h. Presumptive identification of *Staphylococcus* was based on colony morphology on blood agar and mannitol salt agar plates (Oxoid, Basingstoke, United Kingdom), Gram staining, and the detection of enzyme production (coagulase tube test; IBSS Biomed, Poland). A single colony from selected coagulase-positive pure strains was further identified by matrix-assisted laser desorption ionization-time of flight mass spectrometry (MALDI-TOF MS), as previously described^16^. Raw spectra were processed using MALDI Biotyper v.3.1 software (Bruker Daltonik GmbH, Germany). The results were classified using the score values proposed by the manufacturer. The final conformation of each *S. pseudintermedius* isolate was conducted using polymerase chain reaction restriction fragment length polymorphism PCR-RLFP analysis with the primers for the *pta* gene and reaction conditions previously described by Malisova et al.^17^.

### Antibiotic resistance

All isolates of *S. pseudintermedius* were screened for antibiotic susceptibility using both disc diffusion and MIC (minimum inhibitory concentration) methods (Sensititre, *Staphylococcus* MIC plates, Thermo Fisher Scientific, Waltham, MA). Oxacillin MIC was performed for all isolates. Antimicrobial disc diffusion tests included (μg/disc): penicillin G (10), oxacillin (1), amoxicillin-clavulanate (30), erythromycin (15), clindamycin (2), gentamicin (10), tobramycin (10), ampicillin (10), rifampin (5), tetracycline (30), marbofloxacin (5), ciprofloxacin (5), chloramphenicol (30), fusidic acid (10), tigecycline (15), trimethoprim/sulfamethoxazole (1.25/23.75), and linezolid (30) (Antimicrobial Susceptibility Disks, Oxoid Ltd., Wade Road Basingstoke, United Kingdom). Antimicrobial resistance phenotyping of isolates was performed and interpreted according to the Clinical and Laboratory Standards Institute (CLSI)^18^ and CLSI VET08 ED4:2018 (http://vet01s.edaptivedocs.info). Tigecycline and fusidic acid data were interpreted according to the study protocol^19,20^. In cases where isolates from the MIC method exhibited oxacillin resistance, and the presence of *mec*A or *mec*C gene was not confirmed, the nitrocefin biochemical test was conducted (Cefinase test, BioM´erieux Inc.). The double-disc diffusion test (D-test) was performed on all isolates to detect inducible clindamycin resistance.

The presence of genes involved in resistance to various antibiotics was determined using the PCR method and using positive controls, as previously described^21^.The detected genes determinants tesified the potential resistance against penicillin (*bla*Z), aminoglycosides (*aac(6’)Ie-aph(2″)Ia*), β-lactamase (*mec*A, *mec*C), glycopeptides (*van*A and *van*B), macrolide-lincosamide-streptogramin (*erm*A, *erm*B, and *erm*C), tetracyclines (*tet*(K), *tet*(L), *tet*(M) and *tet*(O)), fusidic acid (*fus*B), and mupirocin (*mup*A).

The *S. pseudintermedius* were described as multidrug-resistant isolates at the phenotypic and genotypic level when three or more classes of antimicrobial agents and the presence of three or more resistance genes to different antimicrobial groups respectively were detected.

### Biofilm formation

Strains were tested for slime production by using the Congo red agar (CRA)^22^ method and microtiter plate (MTP) test^23^. *Staphylococcus epidermidis* PCM 2532 was used as the positive control. Bacterial growth on CRA was interpreted using a colorimetric scale^22^. All red shaded colonies were interpreted as non-biofilm producers. Brown shaded colonies were considered to be indicative of weak slime production activity. Black shaded colonies were considered typical slime producer strains. MTP was interpreted following previous descriptions^24^. Biofilm formation ability was considered positive at a threshold of 0.477. Classification criteria were established for positive biofilm formers, whereby: weak biofilm formers: 0.477 < A570 ≤0.954; intermediate positive biofilm formers: 0.954 < A570 ≤1.908; and strong biofilm formers: A570 > 1.908. The MTP procedure was performed three times, with four replicates for each isolate. A standard PCR technique was used for *ica*A^25^ and *bap*^26^ genes, with *S. epidermidis* PCM 2532 and *S. epidermidis* AIR08630 being used as positive controls, respectively.

### MLST and population structure analysis

*S. pseudintermedius* allele sequence types (STs) were determined by multi-locus sequence typing (MLST) using a sequence alignment tool (MEGA X 10.1.). The focus was on seven housekeeping loci described by^27^ and^28^. The ST of each *S. pseudintermedius* isolate was determined using the search tool, with a combination of loci in the *S. pseudintermedius* MLST database^29^. All alleles and isolates with novel combinations of alleles were submitted to the MLST database curator, Vincent Perreten (vincent.perreten@vetsuisse.vbi.unibe.ch). The clonal relationships of the obtained sequence types were connected with entries in the global PubMLST *S. pseudintermedius* database. All entries available at the time of analysis were clustered using the same *goe*BURST procedure database (http://www.phyloviz.net/goeburst/). To determine the clonal relationship between the detected STs and those found in the global Pub-MLST *S*. *pseudintermedius* database, all entries available in April 2021 were clustered using the same goeBURST procedure. Clonal complexes (CCs) were established by matching STs that only differed for one housekeeping gene sequence from the founder genotype—single locus variants (SLVs).

### Statistical analysis

Statistical analysis was carried out using the R statistical package (v3.6.3.). The prevalence and confidence intervals of *S. pseudintermedius* and MRSP were calculated using the bootstrap method. Data on the characteristics of cats, along with their medical history and environmental living conditions, were compared with scores on antibiotic resistance and biofilm formation properties of *S. pseudintermedius* isolates. All data were analyzed using the Shapiro–Wilk test, Wilcoxon test, Kruskal–Wallis test, Chi-square tests, and Fisher's test. *P* < 0.05 was considered statistically significant.

## Results

In total, 35 distinct *S. pseudintermedius* isolates from 27 cats were isolated (GenBank accession numbers of 16S RNA sequences of *S. pseudintermedius* isolates MK681217.1-MK681223.1, MK447569.1-MK447594.1). Isolated *S. pseudintermedius* strains were deposed in the Polish Collection of Microorganisms, Hirszfeld Institute of Immunology and Experimental Therapy, Polish Academy of Sciences, under accession numbers from PCM 3072 to PCM 3106.

*S. pseudintermedius* had a prevalence of 2.49% (95% CI: 1.17–3.99%) and 7.61% (95% CI: 0–16.67%) in healthy and sick cats, respectively. In contrast, MRSP prevalence was 0.12% (95% CI: 0–0.47%) and 2.98% (95% CI: 0–9.52%) in healthy and sick cats, respectively. *S. pseudintermedius* was most frequently isolated from conjunctival sacs and nares (44.44%, each localization). Around half as many bacteria were isolated from the skin and anus (18.52%, each localization). There were no significant differences between the site of *S. pseudintermedius* isolation and health status. Detailed data on cats colonized with *S. pseudintermediu*s and sites of isolation are provided in Supplementary Table 1.

**Figure 1:**
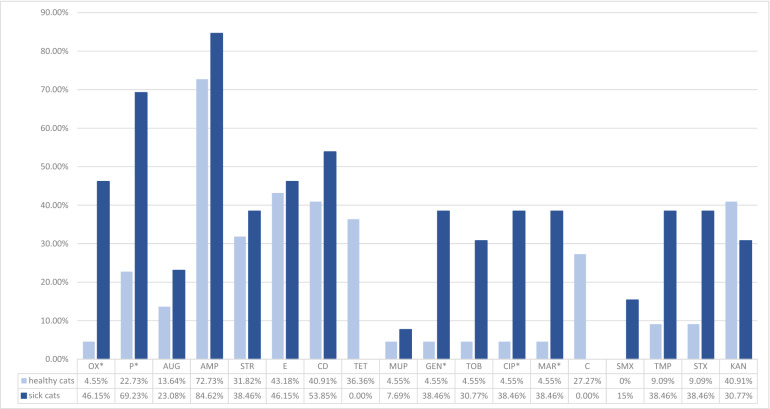
Assimilated data on the antibiotic resistance of *S. pseudintermedius* isolates obtained from healthy and sick cats.

**Figure 2:**
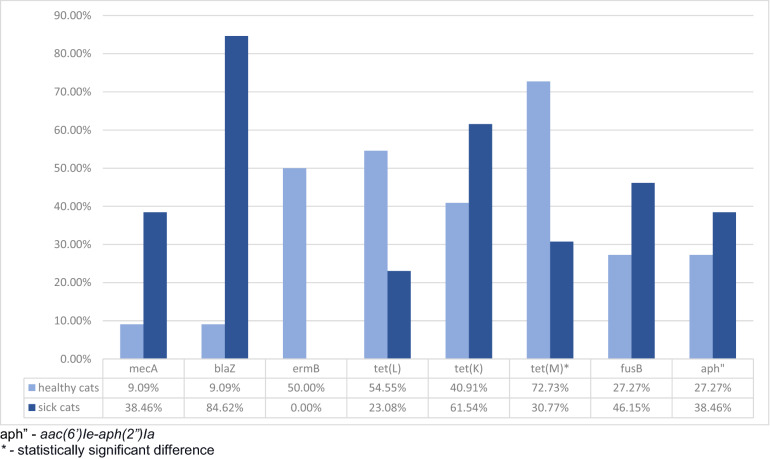
Assimilated data on the antibiotic resistance genes of *S. pseudintermedius* isolates obtained from healthy and sick cats.

No resistance was detected to fusidic acid, vancomycin, quinupristin-dalfopristin, tigecycline, rifampin, and linezolid in isolates collected from healthy and sick animals. No isolates harbored *erm*A, *erm*C, *tet*(O), *mup*A, *van*A, and *van*B genes. Oxacillin resistance was connected with the hyperproduction of beta-lactamase in one isolate. Phenotypic resistance to the three listed chemotherapeutic classes was recorded in 50% and 38.46% of *S. pseudintermedius* isolates from healthy and sick cats, respectively. In comparison, genetic multidrug resistance was detected in 59% and 46.15% of healthy and sick cats, respectively. Almost all isolated MRSPs were multidrug-resistant. Only one MRSP isolated from a sick animal was resistant to oxacillin. Sick animals were more frequently colonized with *S. pseudintermedius* isolates, which were phenotypically resistant; however, these results were not statistically significant. Compared to those of healthy cats, the isolates of sick cats were significantly more frequently resistant to oxacillin (*p* = 0.006; OR = 0.06; CI 95%: 0–0.63), penicillin (*p* = 0.012; OR = 0.14; CI 95%: 0.02–0.76), ciprofloxacin (*p* = 0.019; OR = 0.08; CI 95%: 0–0.9), and marbofloxacin (*p* = 0.019; OR = 0.08; CI 95%: 0–0.9). Phenotypic resistance to tetracycline and chloramphenicol was only detected in the isolates of healthy cats. *Erm*B and, more frequently, *tet*(L) and *tet*(M) genes were detected in the isolates of healthy cats. Only the frequent detection of *tet*(M) was statistically significant (*p* = 0.032; OR = 5.66; CI 95%: 1.08–36.01). Detailed data on phenotypic and genotypic resistance in the isolates of *S. pseudintermedius* are presented in Figs. 1 and 2.

Even though the investigated isolates did not harbor *ica*A and *bap* genes, they had strong biofilm-forming properties. All isolates in MTP showed biofilm-forming properties, whereas three isolates on CRA grew as red shaded colonies, which was interpreted as non-biofilm producers. The results of the phenotypic methods (CRA and MTP) are presented in Table 1.

**Table 1:** Comparing of biofilm-producing properties in CRA and MTP method for *S. pseudintermedius* strains in healthy and sick cats.

Method	CRA			MTP			
	Strains unable to produce slime [%]	Week slime producer strains [%]	Slime producer strains [%]	Strains unable to produce slime A570 < 0.447 [%]	Week slime producer strains 0.447 < A570 ≤ 0.954 [%]	Medium slime producer strains 0.9546 < A570 ≤ 1.908 [%]	Strong slime producer strains A570 > 1.908 [%]
Healthy cats	18.2	54.5	27.3	0	0	59.1	40.9
Sick cats	7.7	53.8	38.5	0	0	46.2	53.8
All cats	14.3	54.3	31.4	0	0	54.3	45.7

CRA—Congo red agar.

MTP—microtitre plate test.

A570—the absorbance signals at a wavelength of 570 nm.

We did not detect any statistical relationship between animal features and more frequent colonization with *S. pseudintermedius* or MRSP in cats. Environmental factors did not influence colonization rates, including the number of household members, owner's medical history, previous hospitalization, or treatment of household members (people and animals). Only the presence of dogs in the same households was a statistically significant factor increasing the frequency of *S. pseudintermedius* colonizing both healthy and sick cats (*p* = 0.006 for both groups).

**Figure 3:**
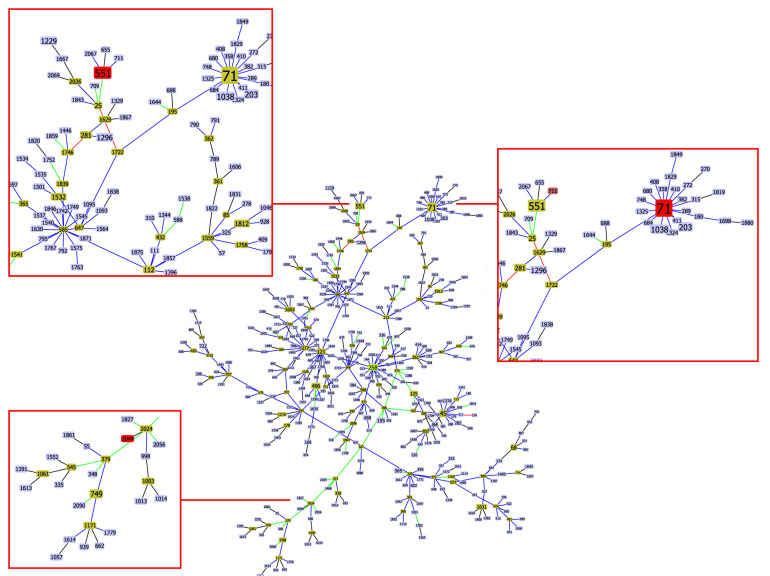
Population snapshot of MRSP in the *goe*BURST analysis. Branches were connected at the single locus variant level to show how STs were related.

STs were determined for 13 isolates from sick cats and 12 isolates from healthy cats. All isolates were submitted to the public database for molecular typing and microbial genome diversity (https://pubmlst.org/bigsdb?db=pubmlst_spseudintermedius_isolates). Two new alleles of *pur*A gene were described (purA86 and purA87). The isolates were assigned to 19 types, of which 16 were submitted with new STs. The most frequently described ST was 551 (four isolates from three cats). We obtained the first records of ST531 and ST551 in cats. MRSP was assigned to ST71, 551, 1884, 2044, and 2048. The relatedness between STs is presented in Fig. 3. The analysis was carried out using the PhyloViz 2.0 software for phylogenetic tree visualisation^30^. Details on STs types are provided in Supplementary Table 1. No leading CCs were detected.

## Discussion

*S. pseudintermedius* is a major contributor to skin and soft tissue infections in pet animals, particularly dogs^4^. Ruscher et al.^31^ showed that MRSP isolates were statistically significantly more frequently isolated from wounds compared to other body parts, especially in dogs. The current study only identified one such case for cats; however, only a small number of cats with wounds were evaluated (n = 8), which means that more data is required. Interestingly, the frequency that *S. pseudintermedius* was isolated from the nares of cats was similar to that of conjunctival sacs. The bacteria occurred in both locations at the same time in just one-quarter of the cats. The observation is important for the population sampling design of future studies. A similar conclusion was obtained in a report on *S. aureus*^32^.

Knowledge remains limited on the characteristics of bacteria occurring in cats, as the prevalence of *S. pseudintermedius* is low. Consequently, the number of investigated isolates is low in cats compared to dogs. The prevalence of *S. pseudintermedius* in cats was previously reported as 2.49–8.8%, while that of dogs exceeded 90%^2–6,33^. Thus, *S. pseudintermedius* might not be a natural microbiota of cats^34^. The previously reported prevalence of *S. aureus* was higher in healthy cats (13.62%)^15^ compared to that obtained for *S. pseudintermedius* in the current study (2.49%). This result indicates that some CPS species (such as *S. pseudintermedius *and *S. schleiferi *spp*. coagulans*) more typically occur in healthy dogs compared to cats^3,35–37^. *S. pseudintermedius* is usually isolated from cats kept with dogs^33^, which was confirmed by the current study. This was also the only factor that had a statistically significant influence on the colonization rate of cats. Other previously identified factors had no impact in the current study, including hospital admissions, surgical interventions, administration of antibiotics, and corticosteroids^7,38,39^.

Selecting effective antimicrobial therapy for animals is becoming increasingly difficult, which, along with the zoonotic potential of *S. pseudintermedius*, is why it is important to monitor phenotypic and genotypic antimicrobial profiles. The current study recorded relatively low MRSP prevalence (0.12% and 2.98% in healthy and sick, respectively); however, the percentage of multidrug-resistant isolates in both groups was of concern. Interestingly, in one isolate, oxacillin resistance was not connected with *mec*A or *mec*C presence, but was caused by the hyperproduction of beta-lactamases. Unfortunately, it is difficult to compare the antimicrobial properties of *S. pseudintermediu*s originating from cats, leading to a low number of isolates being described. Usually, data on the origin of dogs and clinical specimens are documented. As expected, higher resistance was detected in isolates collected from sick animals. Nevertheless, minimal differences existed in the resistance to ampicillin, streptogramin, erythromycin, clindamycin, mupirocin, and kanamycin.

Interestingly, *S. pseudintermedius* isolated from healthy cats was resistant to chloramphenicol and tetracycline. This result corresponded to the frequent presence of genetic determinants of tetracycline resistance in isolates from healthy cats. The widespread presence of *tet*(K) and *tet*(M) in *S. pseudintermedius* isolates was previously described^34^. *S. pseudintermedius* isolates from sick animals were more frequently phenotypically and genetically resistant to beta-lactams, limiting the successful application of antibiotics in treatments. Almost 85% and 23% of isolates in the current study harbored *bla*Z and *mec*A genes, respectively. These findings supported those of Smith et al.^34^. The previous study also showed a high percentage (over 80%) of multidrug-resistant isolates among MRSPs^7^.

Previously described *S. pseudintermedius* isolates were characterized by high biofilm-producing properties^40,41^. Our study also showed that all isolates exhibited high or intermediate slime production characteristics in MPT. However, the most recognized genetic determinants connected with the propagation of slime production, including operon *ica* and *bap* genes, were not detected in any of the investigated *S. pseudintermedius* isolates. A previous study also reported some *S. pseudintermedius* isolates produce slime despite not harboring *ica*A or *ica*D genes. However, this situation was described as an exception rather than a usual phenomenon for *S. pseudintermedius*^41^. Future studies should characterize the mechanism of biofilm production in *S. pseudintermedius*.

MLST typing of *S. pseudintermedius* strains showed that this study first described 72% of STs. ST531, ST551, and ST71 were previously recorded in the PubMLST database; however, only ST71 was previously recorded in cats. ST71 is considered a leading clone of MRSP in Europe (https://pubmlst.org) for dogs, cats, and humans, including both clinical patients and healthy carriers. The presence of ST71 in people is usually connected with previous contact with dogs^42,43^; however, the current study showed that owners of cats might also be at risk of MRSP infection. In particular, Latronico et al.^44^ showed that MRSP ST71 strains of human origin adhered equally well to canine and human corneocytes. Thus, MRSP ST71 might be able to adapt to human skin. Therefore, it is essential to recognize MRSP as a zoonotic pathogen. Similar to previous reports, isolates classified in the current study as ST71 were characterized by multidrug resistance^42,43^. ST551 was previously recorded in dogs and human carriers in Europe and North America (https://pubmlst.org). All reported isolates were MRSPs. Evaluation of MRSPs by Kizerwetter-Świda et al.^45^ documented the emergence of ST551 in dogs in Poland. Consequently, the authors suggested that CC551 might become more prevalent compared to CC71. Of note, ST71 and ST551 were mainly isolated from animals with clinical signs of infection in the current study. However, regardless of whether it originated from healthy or sick cats, all individuals had MRSP and those isolates that were characterized with the highest antibiotic resistance at both the phenotypic and genotypic level. Despite our study identifying 16 new STs, other isolates belonging to CC71 or CC551 were not detected.

In conclusion, this study provided preliminary insights on *S. pseudintermedius* colonization in cats. Previously^15^, differences in *S. pseudintermedius* colonization frequency in healthy and sick cats were detected. The current study characterized *S. pseudintermedius* isolates originating from cats under different conditions. Of importance, both healthy and sick cats groups had higher probabilities of *S. pseudintermedius* colonization when kept with dogs. This finding supports the hypothesis that this bacterium species is more adapted to dogs compared to cats and should not be treated as natural feline microbiota. Knowledge remains limited on how the CCs of *S. pseudintermedius* spread. This issue has led to a low number of typing isolates and low submission rate of typed *S. pseudintermedius* isolates in existing databases. All typed *S. pseudintermedius* isolates mostly belong to CCs previously described in dogs. Only a few strains, like CC71 and CC551, also occur in humans. The ability of MSSPs and MRSPs to acquire and maintain resistance genes, along with the propensity for the horizontal transfer of resistance determinants, represents a potential threat in both veterinary and public health settings.

## Supplementary Information

Supplementary Information 1.

Supplementary Information 2.

## Data Availability

All data are presented in the main paper and in a supporting file.
