# Supplementary material for: Epidemiology of Staphylococcus pseudintermedius in cats in Poland
Source: Sci Rep. 2021 Sep 23;11:18898. doi: 10.1038/s41598-021-97976-z (PMC8460698; doi:10.1038/s41598-021-97976-z)
Supplement: Supplementary file 1 — Supplementary Information 1. [file 41598_2021_97976_MOESM1_ESM.docx]

**Supplementary Table 1.** Detailed data on *S. pseudintermedius* isolates and cats colonized with *S. pseudintermedius*.

| Legend:  OX - oxacillin; P - penicillin; AUG - amoxicillin-clavulanate; AMP - ampicilin; STR - streptogramin; E - erythromycin; CD - clindamycin; TET - tetracycline; MUP - mupirocin; GEN - gentamicin; TOB - tobramycin; CIP - ciprofloxacin; MAR - marbofloxacin; C - chloramphenicol; SMX - sulfmethoxazole; TMP - trimethoprim; STX - trimethoprim/sulfmethoxazole; KAN - kanamycin.  n/a - not applicable |
| --- |
|  |
|  |
|  |
